# Two-stage hip revision arthroplasty with a hexagonal modular cementless stem in cases of periprosthetic infection

**DOI:** 10.1186/1471-2474-15-398

**Published:** 2014-11-26

**Authors:** Ralf Dieckmann, Dino Schulz, Georg Gosheger, Karsten Becker, Kiriakos Daniilidis, Arne Streitbürger, Jendrik Hardes, Steffen Hoell

**Affiliations:** Department of Orthopedics and Tumor Orthopedics, Münster University Hospital, Albert-Schweitzer-Campus 1, A1, 48149 Münster, Germany; Institute of Medical Microbiology, Münster University Hospital, Albert-Schweitzer-Campus 1, A1, 48149 Münster, Germany; Department of Orthopedics, Hannover University Hospital, Anna-von-Borries-Strasse 1-7, 30625 Hannover, Germany

**Keywords:** Two-stage revision, Periprosthetic infection, Hip arthroplasty, Cementless, Modular, Hexagonal

## Abstract

**Background:**

Two-stage revision arthroplasty is today regarded as the gold standard treatment method for deep prosthetic joint infection. The aim of the present study was to evaluate clinical and functional outcomes with the Modular Universal Tumor And Revision System (MUTARS) RS stem in patients undergoing two-stage revisions.

**Methods:**

The functional and clinical outcomes for 43 patients who had undergone two-stage revision procedures for PJI were analyzed in a retrospective study. The minimum follow-up period was 24 months. Shorter follow-up periods were only observed when there were complications such as loosening or recurrent infection. The mean follow-up period was 3.86 years (range 7 months to 11.6 years).

**Results:**

The success rate with infection control for PJI was 93%. Reinfection occurred in four cases (7%). The risk of reinfection after MRSA infection was 20.5 times greater (*P* >0.01) than with sensitive or unknown bacteria. Two aseptic loosening occurred after 7 and 20 months. The average Harris Hip Score was 80 (range 62–93).

**Conclusion:**

This retrospective study showed a 93% rate of eradication using specific antibiotic therapy. With the modular MUTARS RS stem, there was a low rate of aseptic loosening of 4.6%. MRSA infection was identified as a risk factor for reinfection. The two-stage procedure with modular cementless implants used is therefore appropriate for treating periprosthetic infections associated with hip endoprostheses.

**Electronic supplementary material:**

The online version of this article (doi:10.1186/1471-2474-15-398) contains supplementary material, which is available to authorized users.

## Background

The number of total hip arthroplasties (THA) carried out in Germany has increased in recent years up to 160,000 annually [1]. The main reasons for this are the aging of the population and technical improvements in orthopedic surgery. The current rate of prosthetic joint infection (PJI) in Europe and the United States is around 0.6–1.0% for THA [1–3]. This indicates that not only the number of THA increasing, but also the absolute numbers of infections.

Treatment for PJI depends on the duration of symptoms. Early or hematogenous infections, with symptoms for less than 4 weeks, can be treated with local debridement and retention of the implant. In patients with late infections, one-stage or two-stage procedures are necessary [4].

As success rates are above 90%, two-stage revision is the first choice for patients with late PJI in Germany [5, 6]. Either cemented or cementless revision systems can be used [5–9]. Cemented fixation was used for reimplantation in the past, because it allows antibiotics to be used in the cement to reduce the risk of recurrent infection [7, 8]. However, following good results with cementless fixation in aseptic surgery [10, 11], there have been increasing numbers of publications describing successful cementless fixation in two-stage revision procedures [3, 5, 12–17].

The aim of the present study was to evaluate clinical and functional outcomes with the Modular Universal Tumor And Revision System (MUTARS) RS stem, which has been used in our department for more than 10 years, in patients undergoing two-stage revisions.

## Methods

### Patients

The functional and clinical outcomes for 43 patients who had undergone two-stage revision procedures for PJI were analyzed in a retrospective study. The patients had been treated between 2000 and 2012 in a university orthopedic department. We had an ethics approval of the local ethic committee of the University of Münster (2014-324-f-N). Every patient were informed about the study and agreed to publish their data. A consent statement was signed.

They included 21 men and 22 women, with an average age of 66 years (range 40–84 years). The minimum follow-up period was 24 months. Shorter follow-up periods were only observed when there were complications such as loosening or recurrent infection. The mean follow-up period was 3.86 years (range 7 months to 11.6 years). The indication for THA was primary osteoarthritis (OA) in 31 cases, femoral neck fracture in six cases, secondary OA after acetabular fracture in four cases, rheumatoid arthritis in one case, and necrosis of the femoral head in one case. The last operation for the explantation of the prosthesis was in mean 43,5 months (1 months – 24 years).

### Clinical and functional follow-up

All of the patients received radiographic and clinical follow-up examinations. In case of death (n = 2), the last clinical and radiographic examination was evaluated, and inquiries were also made of the patient’s relatives. The Della Valle–Paprosky classification was used to classify femoral bone defects [18]. The functional outcome was evaluated at the outpatient examinations using the Harris Hip Score [19].

### End points and definitions

The inclusion criterion for the patients was a minimum follow-up period of 24 months, or less in case of early loosening of the stem or infection. PJI was diagnosed if at least one diagnostic method was positive in accordance with the Centers for Disease Control criteria [20]. The primary end points of the study were successful treatment for infection or reinfection with loosening of the prosthesis. Clinical cure, with no clinical signs of inflammation and negative C-reactive protein findings, was assessed by the treating clinician at the date of the last available follow-up. Aseptic loosening of the stem was a secondary end point.

### Surgical treatment

**Figure 1:**
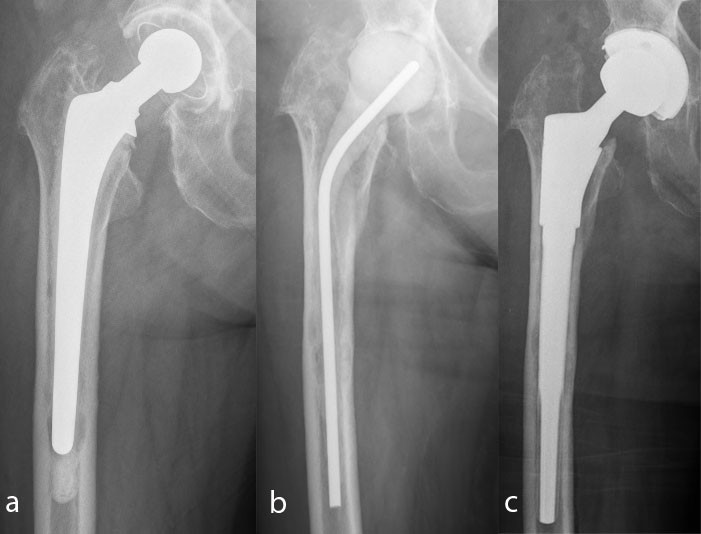
**Two-stage procedure in a 74-year-old-woman. a**: Septic loosening of the hip. **b**: Explantation of the hip prosthesis and spacer implantation. **c**: Reimplantation of a hip prosthesis with the MUTARS RS stem and a cementless cup.

If at least one Centers for Disease Control criterion [20] was positive, a two-stage revision was performed (Figure 1). All of the patients were treated with an antibiotic-loaded polymethylmethacrylate (PMMA) spacer. We collected at least three biopsies for microbiological examination. The composition of the antibiotics in the spacer was adapted to the bacterial resistance (Table 1). Between 2000 and 2004, seven patients were treated with a short period of parenteral antibiotic therapy for a mean of 20 days (range 17–26 days) between both operations. Since 2005, all patients have been treated with parenteral antibiotic therapy for at least 2 weeks, followed by oral antibiotic therapy for at least 4 weeks. If the bacteria involved were not identified, calculated antibiosis with a cephalosporin and clindamycin was administered. In other cases, specific antibiotic therapy was used.

**Table 1:** **Antibiotic combinations in antibiotic-loaded polymethylmethacrylate (PMMA) spacers**

Antibiotic combination	Patients (n)
Gentamicin	11
Gentamicin/vancomycin	4
Gentamicin/vancomycin/clindamycin	16
Gentamicin/clindamycin	11
Gentamicin/clindamycin/flucloxacillin	2

Before replantation we paused the antibiotic therapy for at least two weeks. If the C-reactive protein was less than 2 mg/dl we do the reimplantation. The MUTARS RS stem (Implantcast Ltd., Buxtehude, Germany) was used for reimplantation. It is a modular revision stem with a hexagonal stem design [21] and has included a hydroxyapatite layer since 2006. For acetabular reconstruction we used in three cases a cemented dual mobility cup, in eight cases a cemented polyethylene, in 22 cases a cementless cup and in 10 cases a reconstruction with an antiprotrusio cage. In three cases we used the cage in combination with spongiosa chips of an allograft. After reimplantation, 2 weeks of the parenteral antibiotic therapy was administered, followed by 4 weeks of oral antibiotic therapy.

### Statistical analysis

Statistical analysis was performed with IBM SPSS Statistics for Windows, version 21.0 (IBM Corporation, Armonk, New York, USA).

## Results

### Diagnosis of the periprosthetic infection

The leading indication for explantation was bacteria in the aspiration fluid in 18 cases, fistula in 12 cases, purulent synovial fluid in eight cases, elevated leukocyte counts (>4500) in the aspiration fluid in combination with elevatd C-reactive protein in four cases, and positive leukocyte scintigraphy in combination with elevated C-reactive protein in one case. Microorganisms were identified in 88.3% of cases (Table 2). *Staphylococcus aureus* was present in most cases.

**Table 2:** **Microorganisms identified in soft-tissue samples**

Microorganism	Patients (n)
*Staphylococcus aureus*	13*
*Staphylococcus epidermidis*	12
*Enterococcus faecalis*	3
*Streptococcus gallolyticus*	2
*Corynebacterium*	2
*Escherichia coli*	2
*Staphylococcus haemolyticus*	2
*Enterobacter cloacae*	1
*Neisseria* species	1
*Micrococcus luteus*	1
*Serratia marcescens*	1
*Staphylococcus capitis*	1
*Staphylococcus cohnii* species	1
*Staphylococcus hominis*	1
*Staphylococcus warneri*	1
*Streptococcus agalactiae*	1
*Streptococcus bovis*	1
*Streptococcus pluranimalium*	1
*Peptococcus* species	1
*Propionibacterium* species	1
*Pseudomonas aeruginosa*	1
Sterile	7
Patients with two species	6
Patients with eight species	1

*Two were methicillin-resistant.

### Infection therapy

A spacer exchange and repeated complete PJI management were necessary in four cases. The cause of persisting infection was a nonsensitive spacer in a patient with methicillin-resistant *S. aureus* (MRSA) in one case, persistent fistula in two cases, and persistently high C-reactive protein levels in one case. The mean C-reactive protein level was 0.8 mg/dL (0.5–2.0 mg/dL) at the time of reimplantation.

The success rate with infection control for PJI was 93%. Reinfection occurred in four cases (7%) (Table 3). Another two-stage revision with a cemented prosthesis was carried out in two cases, and resection arthroplasty was performed in two patients. Two patients with reinfection had MRSA infections. The risk of reinfection after MRSA infection was 20.5 times greater (odds ratio; *P* >0.01) than with sensitive or unknown bacteria.

**Table 3:** **Patients with recurrent infection**

Patient no.	Original indication	Risk factors	Revisions	Microorganism	Spacer period (weeks)	Complications	Time to reinfection (months)
1	Secondary coxarthrosis (acetabular fracture)	Fistula, malignancy, diabetes, two-stage revision	Spacer exchange	MRSA	24	–	16.8
2	Primary coxarthrosis	–	Aseptic stem exchange	–	8	–	32.0
3	Femoral neck fracture	–	–	MRSA	77	–	30.0
4	Primary coxarthrosis	Fistula	–	*S. epidermidis*	4.7	–	22.0

MRSA, methicillin-resistant *Staphylococcus aureus.*

### Della Valle–Paprosky classification of the femur and radiographic analysis

The femoral defects were classified using the Della Valle–Paprosky classification [18]. There were nine patients with type II, 30 with type IIIa, and four with type IIIb femoral defects. A MUTARS RS stem was used in all cases. In two cases there was a subsidence of the stem of 5 mm and 3 mm. Both patients had no sign of loosening after a follow-up of three years.

### Comorbidities and risk factors

Comorbid conditions that were documented consisted of obesity (body mass index >30) in 11 patients, diabetes mellitus in eight patients, malignancy in five patients, long-term cortisone therapy in three patients, endocarditis in two patients, chronic obstructive lung disease in two patients, osteomyelitis of the femur in one patient, PJI of the contralateral knee endoprosthesis in one patient, hepatitis B in one patient, hepatitis C in one patient, and long-term methotrexate therapy in one patient. Four patients had more than one comorbidity.

The number of surgical procedures that had been carried out before the two-stage procedure was also documented as an additional potential risk factor. A two-stage procedure had already been performed in three patients; the infection occurred after the primary operation in 18 patients; 16 patients had one aseptic revision, five patients had two aseptic revisions, and one patient had one septic revision with retention of the prosthesis.

### Complications unrelated to infection

**Figure 2:**
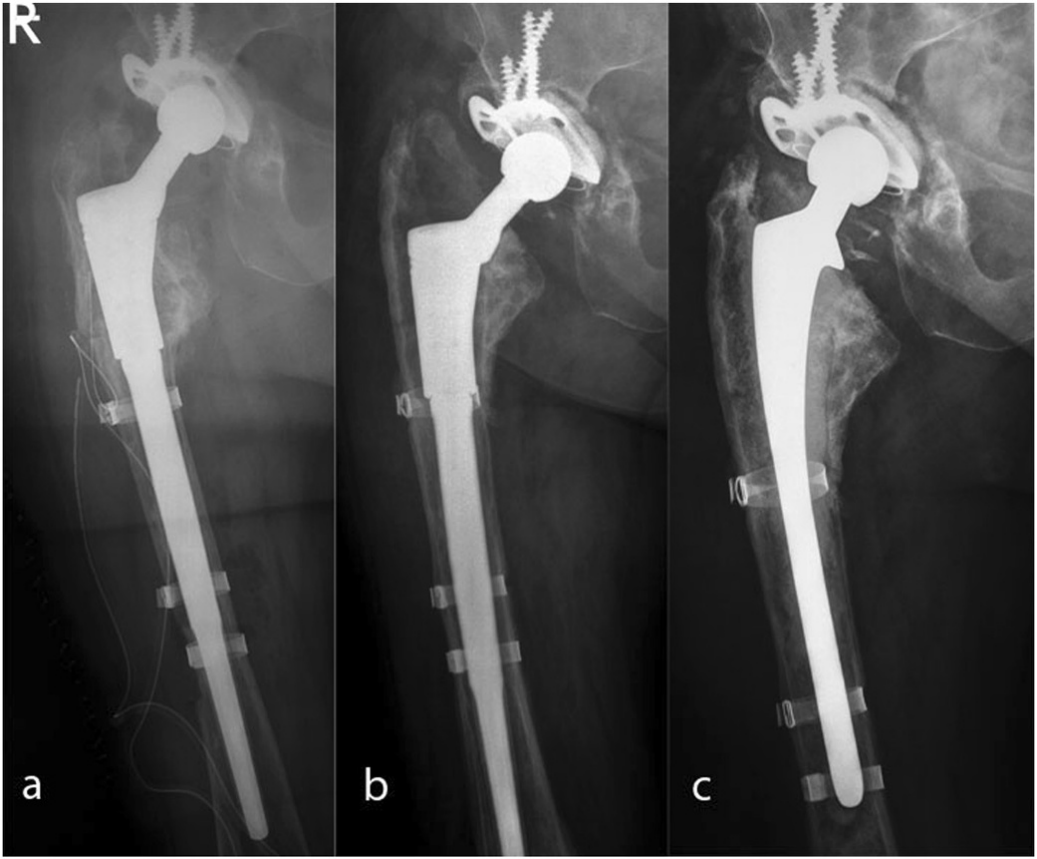
**Aseptic loosening of a MUTARS RS stem in case of a Paprosky IIIa defect.** **a**: postoperative x-ray. **b**: 18 months x-ray control with aseptic loosening. **c**: Revision with a cemented stem.

Two complications associated with the stem occurred. Both involved aseptic loosening after 7 and 20 months, respectively, with a type IIIa Della Valle–Paprosky defect in one case and a type IIIb defect in the other (Figure 2). The preoperative C-reactive protein findings were negative and no bacteria were found in the intraoperative tissue samples. Revision surgery with a long cemented stem was carried out in both cases.

Wound healing problems occurred in four cases. Healing followed after local debridement in all of these patients. There was a trend for patients with diabetes mellitus to have a 4.5 times (odds ratio) greater risk of developing wound healing problems. One patient developed peroneal paralysis postoperatively; immediate revision surgery with shortening of the femoral head was necessary. One patient had a periprosthetic fracture after a fall; osteosynthesis was carried out successfully. Another patient developed dislocation of an inlay, and the cup had to be exchanged. One patient developed recurrent dislocation after reimplantation; the inlay was changed to a 10° insert and the antetorsion of the stem was altered. Another patient had aseptic loosening of an antiprotrusio cage, and an exchange was necessary (Table 4).

**Table 4:** **Patients with complications unrelated to the infection**

Complications	Patients (n)
*Wound healing problems*	4
*Aseptic loosening of the stem*	2
*Recurrent dislocations*	1
*Aseptic loosening of the cup*	1
*Periprosthetic fracture after collaps*	1
*Peroneal palsy*	1
*Inlaydislocation*	1

### Functional outcome

Evaluation of the postoperative Hip Society Score was possible in 37 cases. Aseptic or septic exchanges of the stem had already been performed in three cases already, two of the patients had died, and it was not possible to contact the patient for the questionnaire in one case. The average Harris Hip Score was 80 (range 62–93). Excellent results were seen in four cases, good results in 19, fair results in 11, and poor results in three cases. There was no difference in the functional outcome depending on the bony defect.

## Discussion

Two-stage revision arthroplasty is today regarded as the gold standard treatment method for deep prosthetic joint infection [22, 23]. However, there has been debate on whether cemented or cementless reimplantation is better, in view of the lack of local antibiotic protection with the latter method [5–7, 9, 12–15, 24–26]. In earlier studies on cementless two-stage revision, high rates of loosening and reinfection were reported [25, 26]. However, more recent studies have described good eradication rates and good prosthetic survival [5, 6, 14, 24]. The present study focuses on the reinfection rate and early loosening of the MUTARS RS stem (Implantcast Ltd., Buxtehude, Germany).

Limitation of this study are the retrospective nature of the study and the relatively short follow-up period of 2 years in a few cases. However, most cases of reinfection occur in the first few years after reimplantation [15, 24] and most studies on cementless two-stage revision have reported follow-up periods of 2 years [5, 6, 12, 24, 25]. Subsidence of the stem and early loosening of the components would also occur within the first few years [27–30].

The eradication rates achieved ranged from 82% to 100% with cementless two-stage revisions [5, 6, 12–15, 17, 24–26] and from 84% to 100% with cemented two-stage revisions [31–35]. The eradication rate observed in the present study was 93%, including multiresistant bacteria. The treatments administered in other studies vary widely. Haddad et al. [6] used a course of 5 days of parenteral antibiotics, followed by oral antibiotics for 3 weeks in the interval up to reimplantation, with an eradication rate of 92%. Other studies have reported administering intravenous antibiotics for up to 6 weeks [5, 12–14, 24]. Another limitation in the present study is that we had two different treatment protocols. Initial we treated seven patients in mean only 20 days parenteral between both operations. We had no reinfection with this kind of treatment. To our knowledge there is no study describing the gold standard for the perioperative treatment [23, 36]. Since 2004 we treated with a periods of 2 weeks with parenteral antibiotic treatment and 4 weeks of oral antibiotic therapy were used in the interval to reimplantation, followed by a further course of antibiotic therapy with 2 weeks of parenteral treatment followed by 4 weeks of oral treatment after reimplantation. Fink et al. reported a 100% eradication rate with non-multiresistant bacteria using this sequence [5]. The 2-week period of parenteral antibiotic therapy appears to be short, but it is consistent with the recommendations of Zimmerli et al. [4, 37]. The 3-month period of antibiotic treatment is also in agreement with the recommendations [4, 37].

Local methods of treatment for PJI also vary widely. In earlier studies, resection arthroplasty [25, 26], antibiotic beads [6, 12], and articulating spacers [15] were used. Gentamicin, alone or in combination with vancomycin, was often used [6, 12]. However, it was not possible to successfully treat gram-negative bacteria in particular with this combination. Masri et al. and Fink et al. were the first to describe specific local antibiotic therapy [5, 24], which makes it possible to treat resistant bacteria successfully, on the one hand, while on the other the development of new resistance can be avoided. Specific antibiotic therapy was therefore always used in the present study when possible. In cases of unknown bacteria, gentamicin alone was used. The results, with an eradication rate of 93%, provide support for this form of local therapy.

Multiresistant bacteria such as methicillin-resistant *S. aureus* (MRSA) are an increasing problem [38] and are associated with higher reinfection rates [39]. Kilgus et al. reported that the reinfection rate in cases of multiresistant *S. aureus* and *S. epidermidis* has increased from 19% to 52% [40]. Lim et al. compared 24 patients with resistant microorganisms with 13 patients with nonresistant microorganisms [41] and noted a recurrence rate of 33% in the resistant group, in comparison with no failures in the nonresistant group. Similar results were observed in the present study. Only two patients had MRSA infections, but reinfection occurred in both cases. One had already undergone a two-stage revision due to MRSA infection. The reinfection rate was significantly higher in patients with MRSA infection.

Aseptic loosening occurred in 4.6% of the cases. In a review, Wirtz et al. reported mean loosening rates of 4.4% with cementless stems in cases of aseptic revision and 21.2% with cemented stem revisions [42]. Dohmae et al. [43] demonstrated that there is a 70% lower shear force capacity in revision cases in comparison with primary implantations. This may be the reason why the cement does not fuse with spongy bone. It is therefore necessary for the revision implant to bridge the bone affected by the original femur implant. In two-stage revisions, Nestor et al. [25] reported an 18% rate of loosening using a nonmodular, proximally coated stem, while other studies have reported loosening rates of 0% using nonmodular or modular stems [5, 6, 12–14, 17, 24]. Most of the studies do not report any classification of the defects. The loosening rates observed with two-stage revisions are equal to or better than those with aseptic revisions [42]. It was therefore concluded that the loosening rate depends on the defects involved, rather than on the two-stage procedure.

Other risk factors have also been identified that have an unfavorable influence on the course of salvage procedures following infection. Particularly with primary hip arthroplasty, these factors include postoperative osteoarthritis, multiple surgical revisions, cutaneous and urinary tract infections, chronic liver disease, inadequate antibiotic prophylaxis, and malignancies [44]. Further risk factors include rheumatoid arthritis, steroid therapy, diabetes mellitus, immunosuppressant therapy, and nosocomial infections [45]. The only risk factor for reinfection identified in the present data was the presence of multiresistant bacteria. In fact, the same risk factors appear to be present with two-stage revisions as in primary arthroplasty. It is possible that the number of patients included was too small for statistical significance to be reached.

## Conclusions

This retrospective study showed a 93% rate of eradication using specific antibiotic therapy in accordance with the recommendations made by Zimmerli et al. [4] and Trampuz et al. [46]. With the modular MUTARS RS stem, there was a low rate of aseptic loosening of 4.6%. MRSA infection was identified as a risk factor for reinfection. The two-stage procedure used with the modular cementless implants is therefore appropriate for treating periprosthetic infections associated with hip endoprostheses.

## Authors’ original submitted files for images

Below are the links to the authors’ original submitted files for images. Authors’ original file for figure 1 Authors’ original file for figure 2 Authors’ original file for figure 3 Authors’ original file for figure 4 Authors’ original file for figure 5 Authors’ original file for figure 6
