# Supplementary figures and images for: Two-stage hip revision arthroplasty with a hexagonal modular cementless stem in cases of periprosthetic infection
Source: BMC Musculoskelet Disord. 2014 Nov 26;15:398. doi: 10.1186/1471-2474-15-398 (PMC4289174; doi:10.1186/1471-2474-15-398)

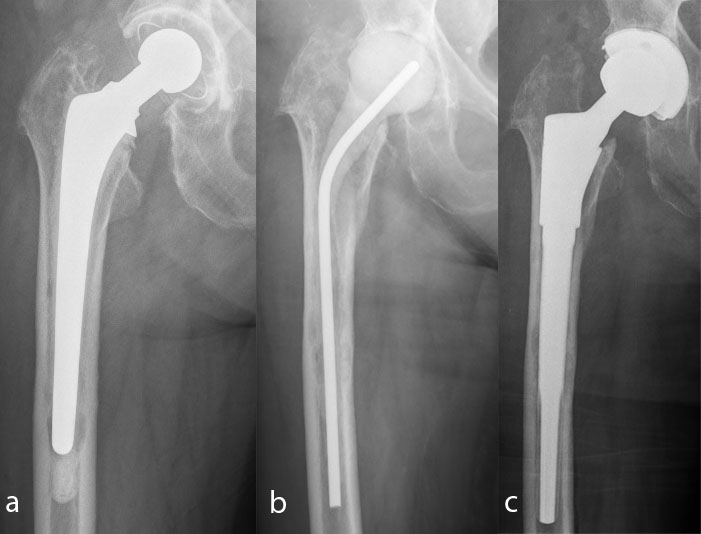

Supplement: Supplementary file 1 — Authors’ original file for figure 1 [file 12891_2014_2370_MOESM1_ESM.jpg]

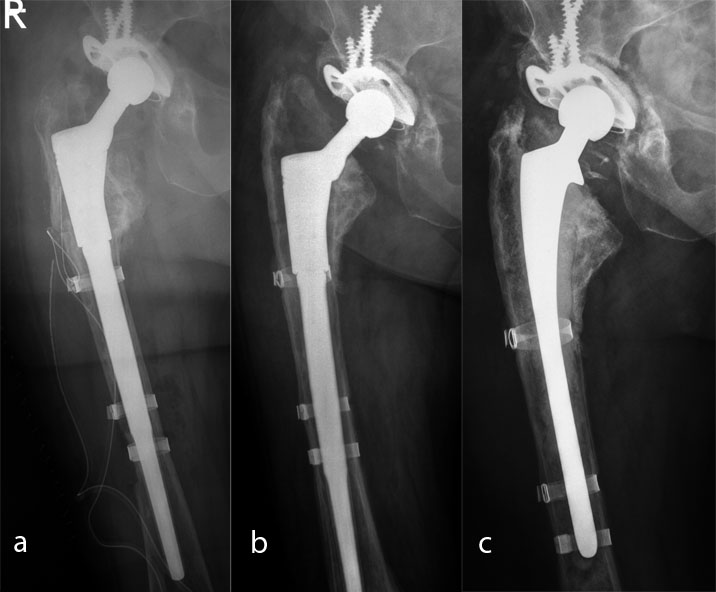

Supplement: Supplementary file 2 — Authors’ original file for figure 2 [file 12891_2014_2370_MOESM2_ESM.jpg]

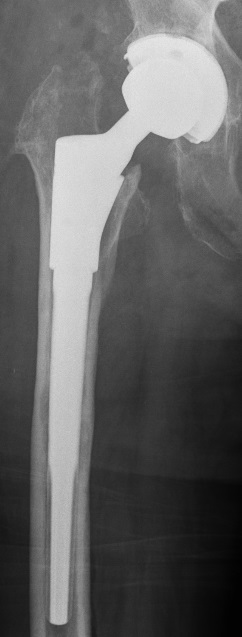

Supplement: Supplementary file 3 — Authors’ original file for figure 3 [file 12891_2014_2370_MOESM3_ESM.jpeg]

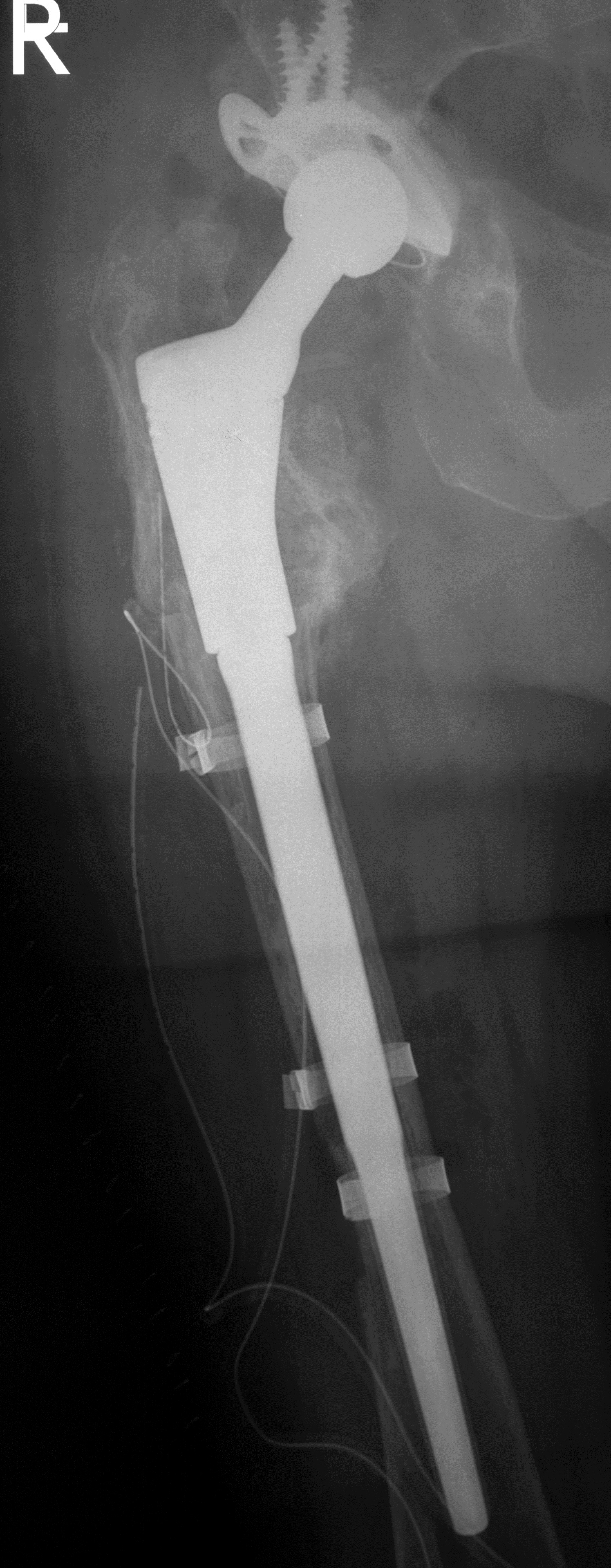

Supplement: Supplementary file 4 — Authors’ original file for figure 4 [file 12891_2014_2370_MOESM4_ESM.jpeg]

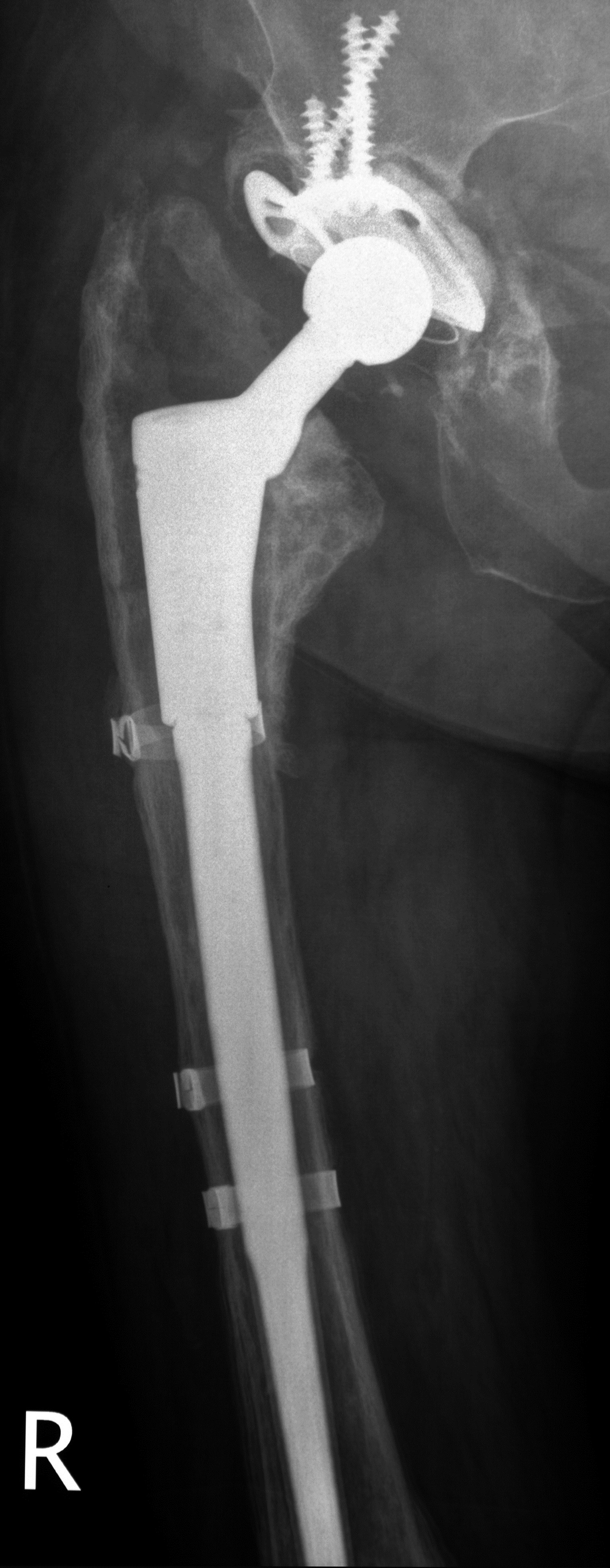

Supplement: Supplementary file 5 — Authors’ original file for figure 5 [file 12891_2014_2370_MOESM5_ESM.jpeg]

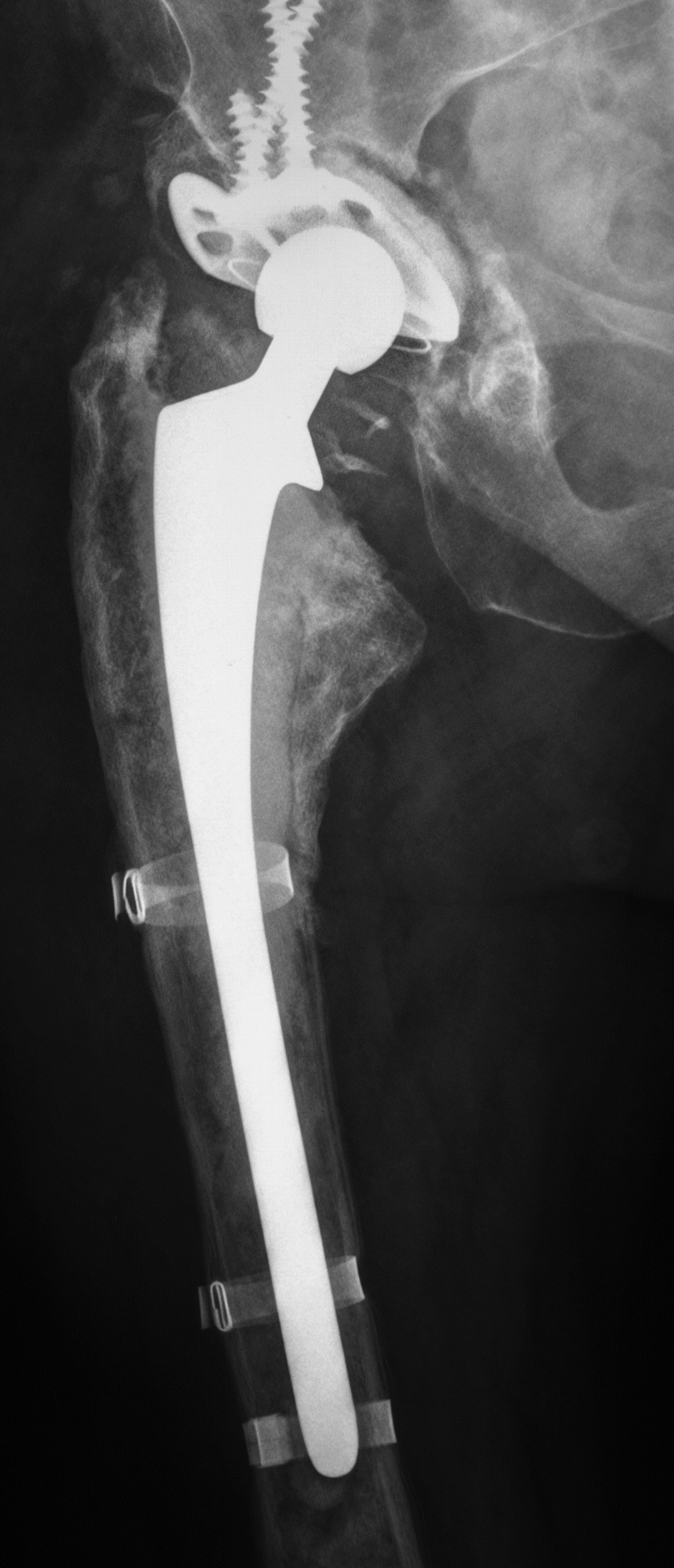

Supplement: Supplementary file 6 — Authors’ original file for figure 6 [file 12891_2014_2370_MOESM6_ESM.jpeg]
